# The Circular Rebound Tool: A tool to move companies towards more sustainable circular business models

**DOI:** 10.1016/j.rcradv.2023.200185

**Published:** 2023-12

**Authors:** Ankita Das, Jan Konietzko, Nancy Bocken, Marc Dijk

**Affiliations:** Maastricht Sustainability Institute, School of Business and Economics, Maastricht University, Tapijn 11 Building D, P.O. Box 616, 6200 MD Maastricht, The Netherlands

**Keywords:** Circular economy, Circular business models, Environmental impact, Business model tool, Rebound effects

## Abstract

Companies design circular business models through experimentation. However, most companies do not consider the environmental impact of their new business model ideas during experimentation, an iterative phase of high uncertainty. Previous research shows that companies typically use ‘rules of thumb’ to estimate environmental impact in this stage due to limited time and reliable information to guide decision-making. This might prevent innovators from detecting unintended rebound effects that offset positive environmental gains of new business models. To mitigate this and let innovators think more profoundly about rebound effects during the circular business model experimentation phase, we propose an evidence-based business model ideation tool, the Circular Rebound Tool, designed around lifecycle thinking, the zero-waste hierarchy, and increased rebound effects awareness. The tool's development follows the design science research method, undergoing continuous improvement through 15 workshops. Our tool can help business innovators gain insights into the environmental impact of their early-stage business ideas.

## Highlights

• Circular business models can lead to unanticipated negative environmental impacts.
• Potential negative rebound effects can be avoided in early design phase of business models.
• Our tool helps designers learn about environmental impacts in the early design phase.
• The tool was tested with designers to develop better circular business models.

## 1 Introduction

Businesses are increasingly setting higher climate targets, as recognition of the urgency to change the current linear take-make-waste system grows (European Commission, 2020). One way in which businesses move towards reducing their potential impacts on climate, biodiversity, land use and resource depletion is by adopting circular business models (Bauwens et al., 2020). When designed the right way, the more service-orientated business models have been suggested to achieve environmental impact reductions of 50–90 %, compared to just selling a product (Tukker, 2004; 2015). Yet in reality, such positive impacts have scarcely been observed or measured, except by a handful of studies. Bocken et al. (2018) identified a reduction of 25 % in environmental impact in the use phase of a pay-per-use business model for washing machines. Fidan et al. (2021) found 50–98 % environmental impact improvements of denim produced using recycled cotton and energy from a combined heat and power plant instead of grid electricity. Another study by Kaddoura et al. (2019) showed that adoption of a service-based business model for passive durable products like event tents, lockers, and recycling bins, can have a 45–72 % reduction of environmental impact by extending product lifetime and durability. However, most studies are often about possible scenarios of new hypothetical business model ideas rather than the real implementation (e.g., Lieder et al., 2017a, 2017b; van Loon and Van Wassenhove, 2018).

There are many strategic motivations for pursuing circular business models, for example, to reduce the reliance on manufacturing from new raw materials, mitigate risks of supply chain disruptions, avoid price volatility, and improve competitiveness (Ali et al., 2017; Alonso et al., 2007; Erdmann and Graedel, 2011; Rosenau-Tornow et al., 2009). Furthermore, companies can stand to benefit from experimentation with circular business models, as it could help them live up to future environmental legislation. There are substantial benefits to early adaptation to these regulations, since the cost of adapting later could be much higher. Hence, there are many reasons why companies have started to trial these new circular business ideas through conducting business model experiments, despite lack of well-documented evidence of environmental impact reductions (Antikainen and Valkokari, 2016a). Business model experimentation is the iterative process of trialling new business innovations, before large-scale implementation (Bocken and Snihur, 2020). Such experiments can help collect data to reduce uncertainty, validate ideas and make evidence-based decisions (Blank, 2013; Bocken et al., 2016b; Konietzko et al., 2020).

But not all circular business models have the same potential to reduce environmental impacts (Tukker, 2004; Zink and Geyer, 2017). Furthermore, many well-intended circular business ideas can be subject to circular economy rebound effects leading to higher consumption of resources. This refers to the unintended consequence where resource efficiency measures, such as waste reduction or energy efficiency, are offset by an increase in consumption or production, resulting in less environmental benefits than expected. (Zerbino, 2022; Zink and Geyer, 2017). Previous studies have suggested that 80–90 % of the environmental impact of products is determined in the design phase itself (McAloone and Bey, 2009; Millet et al., 2007; Tischner and Charter, 2001). Drawing on this and the work by Tukker (2004, 2015) and Mont (2002, 2004) who argue that the environmental benefits of product-service combinations are determined by their design, we argue that the same might be true for the design of new circular business models as novel combinations of products and services. Therefore, many circular economy rebounds (henceforth only referred to as rebound effects) can also get locked-in at this stage and diminish the expected environmental gains. As circular business models become increasingly mainstream, it is important to be able to scrutinize their true environmental impact reduction potential. Although some rebounds may lie outside the direct zone of control of many individual businesses it is crucial to factor in possible rebounds and other unintended consequences in the design and experimentation stage before they are fully deployed, so that missed opportunities and rebound effects can be avoided (Das et al., 2022; Manninen et al., 2018; Sassanelli et al., 2019).

Previous work has found that innovators in companies often do not measure the impact of the new circular business models that they are trialling (except perhaps with rules of thumb, which might be inaccurate). But they are highly interested in creating more positive outcomes, having gone through significant experimentation (Baldassarre et al., 2020; Das et al., 2022). Experimentation is characterised by high uncertainty but also high opportunity to make an impact. It is therefore important to pay more attention to the environmental impact during the early stage of business model design (Bocken et al., 2016b; Bocken et al., 2021; Manninen et al., 2018).

While various circular business model tools exist, there is a need for a tool that can reduce the impact uncertainty during early experimentation, and nudge innovators towards circular business models with lower environmental impact. Through this study, we aim to help practitioners scrutinize their assumptions about the environmental impact reduction potential of their new business model ideas. Business innovators often do not have access to life cycle analysis data, they cannot predict what will happen in the use phase, and they do not know how consumers will change their behaviour based on the new business model (Das et al., 2022). To mitigate this uncertainty, we introduce the Circular Rebound Tool, an ideation decision guide for business model designers with basic knowledge of circular economy and sustainability. The main guiding research question of this study was: *How can companies be supported in preventing rebound effects in the early design phase, in order to create circular business models with lower environmental impact?*

The findings of this study (the Circular Rebound Tool) can be useful for practitioners, think-tanks, decision-makers, academics, and businesses that are experimenting with circular business models. In practice by aiding the development of more sustainable circular business models, and in theory by helping to map potential rebound effects of circular business strategies, and suggesting ways to overcome these. Below, Section 2 gives a conceptual background to the topic, Section 3 describes the method, Section 4 showcases the results, and Section 5 and 6 provide the discussion and final conclusion.

## 2 Conceptual background

In the following subsections, we discuss how companies experiment with new circular business models through business model experimentation. We highlight some of the potential rebound effects or unintended negative consequences from this process, and then discuss the current state of environmental impact forecasting tools in practice and the need for a new tool that can act as an ideation decision-guide.

### 2.1 Experimentation towards circular business models

Circular business models can be of many types (Antikainen and Valkokari, 2016b; Lüdeke‐Freund et al., 2019). A recent review by Pieroni et al. (2020) identified 20 different circular business model archetypes, and 63 sub-types. With so many potential strategies, companies first conduct business model experiments to validate their new business model ideas (Bocken et al., 2016b). The concept of experimentation originates from natural sciences and economics (Bocken et al., 2016b). But, business model experimentation refers to innovative, small experiments that are conducted to trial new business ideas in relatively controlled environments before large-scale implementation (Bocken et al., 2016b). Stakeholders in business often tend to be wary of changing the way their products capture value, in order to not confuse customers and lose sales. Experimentation allows for mistakes and troubleshooting in a more controlled environment, test initial assumptions about a new offering or idea and guide the new business model design by gathering evidence around its execution. For example, field experiments conducted by Poortinga and Whitaker (2018) on reusable cups in cafes showed that offering reusable cups as an option had no negative effect on sales. In fact, sales increased during the experiment. In another example Vandenbroele et al. (2019) confirmed through experimentation in a retail store that presenting meat substitutes next to meat products in the butchery can increase consumer uptake/sales amongst potential non-users, without having any negative effects on the bottom line.

Experimentation differs from business model innovation in that ‘innovation’ refers to the process of operationalising new ways of doing business. Whereas business model experimentation is a means to achieve business model innovation (Foss and Saebi, 2018). We use the term ‘experiments’ as opposed to pilots or trials to emphasise that it is an open-ended process of learning by doing. This concept is already being more widely accepted in practice through methods like the ‘Lean Startup’ (Ries, 2011), effectuation (Sarasvathy, 2008), and design thinking (Peffers et al., 2007), which emphasises an iterative ‘build-apply-measure-learn’ process.

However, when experimenting with circular business models, practitioners can find it hard to keep track of the net environmental impacts of their design decisions (Baldassarre et al., 2020; Das et al., 2022; Manninen et al., 2018). This is due to the high levels of uncertainty surrounding the product or business offering in the experimentation phase. But circular business models are not necessarily more sustainable by default and can be subject to rebound effects (Zink and Geyer, 2017). So it is important to be aware of the true environmental impact reduction potential in the early stages of business model experimentation, to prevent unintended consequences and lost opportunities.

### 2.2 Environmental impact of circular business models and rebound effects

Circular business models have the potential to significantly reduce the environmental impacts of products, but that is not a given (Blum et al., 2020). Well-intended sustainable offerings can lead to unintended negative rebound effects by increasing consumption (Catlin and Wang, 2013; Zink and Geyer, 2017). Further, customers might mistreat a product that is for rent because they perceive a lack of ownership and lower responsibility towards the product (Tunn, 2020), leading to higher replacement, repair or refurbishment rates. Rebound effects are the difference between the expected and actual environmental savings from efficiency improvements, once larger economic considerations have been accounted for (Zink and Geyer, 2017). The phenomenon was first observed in the 1860s in economics and energy efficiency literature, where energy efficiency savings are offset by increased net resource consumption (Jevons, 1866). The concept was then called the ‘Jevons paradox’. It was more recently repopularised by economists Khazzoom and Brookes (Saunders, 1992), who put forth the idea that energy efficiency paradoxically leads to increased energy consumption. The most widely cited example is that of the fuel-efficient car: the cost savings from owning a more fuel-efficient vehicle can be offset by consumers driving more kilometres on average (Druckman et al., 2011) or buying vehicles with more power and weight (Pardi, 2021).

Rebound effects can be classified into four types: i) direct rebound, which refers to the immediate increases in demand resulting from lower prices due to increased efficiency; ii) indirect rebound or secondary effects, which increase the demand for other goods resulting from increased savings from the efficiency of the first good; iii) economy-wide effects, refer to more unpredictable market-wide changes on prices and demand of other goods, resulting from increased efficiency of one good; and iv) transformational effects, which is the ability of increased efficiency to alter consumer preferences, regulations, societal norms and/or technological advancements (Greening et al., 2000). An example from the circular economy is when increased emphasis on recycling could lead consumers to use more disposable products. This was confirmed by the wastepaper recycling experiment conducted by Catlin & Wang (2013), where consumers used more paper towels when presented with a recycling bin.

In one of the first papers to discuss ‘circular rebound’, Zink & Geyer (2017) identified two mechanisms of rebound effects in a circular economy: insufficient substitutability, and price effects. The first assumes that not all secondary goods (recycled, remanufactured, and reused materials) can efficiently substitute primary goods (virgin materials). This means that they at least partly add to what was there earlier, instead of replacing it. The second assumes that circular products are cheaper, which can increase demand and lead to more production of primary and secondary goods, increasing overall material consumption - an effect that could be especially difficult for an individual business to predict and offset. These two mechanisms can lead to circular products simply increasing the size of the pie, instead of taking a share from the space occupied by linear products. An example of this is bicycle/e-scooter graveyards that have resulted from the boom of sharing mobility (Haas, 2017). Many companies have overproduced the number of products by overestimating their market potential which they abandon when going bankrupt (Pyzyk, 2019). Further, the products are often subject to significant wear and tear, vandalism and mechanical breakdown, and at times the lifespan of these devices is only three months (Schellong et al., 2019). This can cancel out any expected environmental impact gains from consumers using less cars for their commute.

More recently, Castro et al. (2022) and Metic & Pigosso (2022) have conducted systematic literature reviews on the circular economy rebound effect and showed that more integration of the impacts of rebound effects into circular business models is still missing. Further, firms need to care about potential rebound effects in order to ensure that they are future proof from upcoming environmental legislation that could require them to include more detailed aspects of their environmental impacts. Another reason could be to avoid greenwashing, legislation to avoid this is for example forthcoming in the UK and EU (Arbinolo, 2023; Ungoed-Thomas, 2023). Further, experimenting with and implementing a new circular business model might be an expensive endeavour, so it makes sense to try to get the initial proposition right from the beginning, rather than having to pivot ex-post. It is therefore important to be aware of the environmental impact of new circular business models during experimentation.

### 2.3 Need for environmental impact forecasting

Several tools and methods exist to measure the environmental impacts of new products or business models. They can be classified into two overarching categories: ex-ante and ex-post tools (Bailey et al., 2002). Ex-ante tools estimate the environmental impact before an idea is implemented, giving innovators the chance to improve their ideas before implementation (Kravchenko et al., 2019; Pojasek, 2009). Examples of this can be the Climate Impact Forecast Tool and Idemat by Delft University (Idemat, 2023; Impact Forecast, 2023). On the other hand, ex-post assessment tools measure the environmental impact of the outcomes after a strategy or plan has been carried out. Common examples of these are the Life Cycle Assessment, Mass-Flow Analysis and the Global Reporting Initiative. The scope of these tools can be focused on the micro, *meso* and/or macro levels (Harris et al., 2021; Johnson and Schaltegger, 2020), referring to the levels of the product or company, industrial networks, and that of cities, nations or society respectively. However, many such tools developed in academia are often not used in practice as they are not tested and co-created with their intended users (Baumann et al., 2002; Tyl et al., 2015).

Past studies have found that practitioners deprioritize tracking the impact in the design and experimentation phase, due to a focus on other aspects of the business model, like the customer desirability or the business case. Baldassarre et al. (2020) conducted a series of sustainable business model ideation workshops with early-stage startups. They found that while all startups started with the idea of building a business model with positive environmental gains, most struggled to measure this environmental impact change through the design process. Often, measuring environmental impact took a backseat when the startups started being more engaged with how the business idea would generate revenue. The startups had to be nudged by the researchers to include more concrete sustainability metrics, and often treated sustainability more as an abstract driver than a necessary condition (Baldassarre et al., 2020).

Research by Das et al. (2022) also showed that both startups and incumbent firms often resort to using rules of thumb to measure the environmental impact of new business ideas during early experimentation. Rules of thumb are heuristics that can, for example, involve the use of internal guidelines or policies regarding circular design, rough estimates of consumer behaviour or the environmental impact reduction using key performance indicators. They choose these over more robust environmental impact measurement methods like the life-cycle assessment or the mass-flow analysis because of the high amount of data and time commitment required for these methods, which is scarce in the uncertain ideation phase. But using them for environmental impact measurement could lead to inaccurate measures. So, while early experimentation can have a big impact on the successful performance of the business model, the environmental impact is often not forecasted or validated.

Further, practitioners can struggle to find a business model that addresses desirability, viability, feasibility and sustainability into account simultaneously (Baldassarre et al., 2020). Often it is not easy to pick and predict the business model that has the most environmental gains in practice. Even the most sustainability-minded businesses must factor in other parameters such as long-term costs, ease of implementation, willingness amongst customers to change, and commercial attractiveness to create successful business models (Figge and Hahn, 2012; Hahn et al., 2010).

Previous research has developed tools for ideation and implementation of circular and sustainable business models as documented in the review by Pieroni et al. (2019). But the tools identified in this review often disregard the environmental impact of these new business models they are helping ideate. More recent studies have developed tools that incorporate environmental impact into business model ideation, however, they fail to account for avoiding rebound effects that might offset any environmental impact reductions (Bjørnbet and Vildåsen, 2021; Nußholz, 2018; Pieroni et al., 2021). Further, to the best of our knowledge, there is no tool that helps practitioners in dealing with possible rebound effects in the experimentation phase that new circular business models might cause. It is therefore necessary to create a tool that can nudge practitioners in the experimentation phase towards circular business model ideas with higher environmental impact reduction potential. Nudging involves using choice architecture to improve users’ decision-making and alter behaviour without restricting options (Thaler and Sunstein, 2008). This can allow practitioners to consider and avoid possible rebounds from the beginning and achieve the most environmental impact gains (Das et al., 2022).

Consequently, we contribute ‘The Circular Rebound Tool’ that nudges users to scrutinize their assumptions about the environmental impact reduction potential of their new circular business model ideas, and nurtures further experimentation towards high-impact circular business models. The tool is designed for use by practitioners with basic knowledge of circular economy without expert guidance. To ensure its quality and relevance, it is developed from literature and practice, and has been tested with relevant user groups as suggested by research on effectiveness of circular business model tools (Bocken et al., 2019). In the following, we explain the design process for the tool.

## 3 Method

**Fig. 1:**
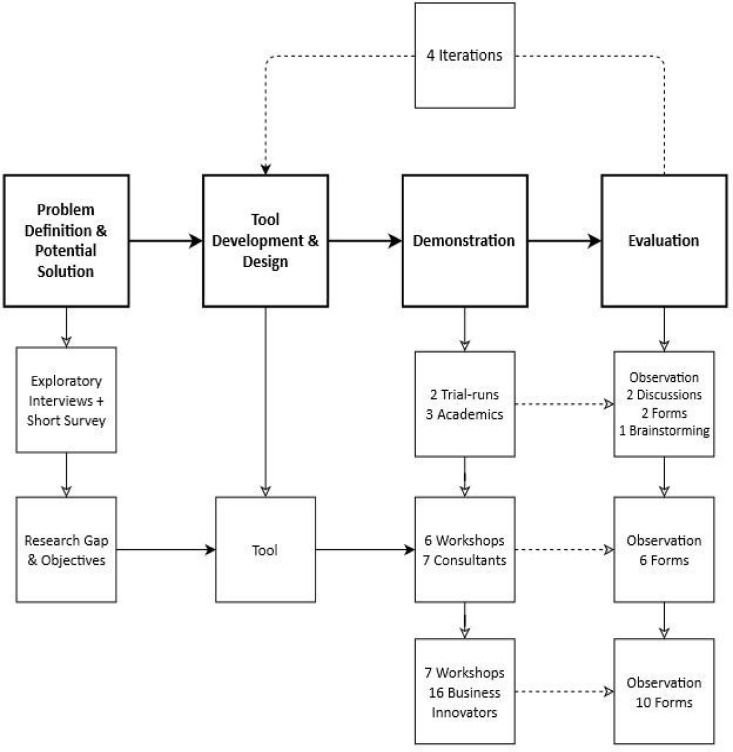
Overview of the DSR method used in this research (adapted from Baldassarre et al., 2020).

This research followed the design science research (DSR) methodology (Peffers et al., 2007), a problem-solving framework (vom Brocke et al., 2020) that fit the needs of this study well, which were to create a tool that would act as an ideation decision-aid for circular business innovators, and improve it further through empirical testing. The DSR method consists of an iterative process of evaluation and improvement of a model or artefact, through application in practice (Carstensen and Bernhard, 2019). The process constituted of four condensed steps – based on the 7 steps detailed by Baldassare et al. (2020) – demonstrated in Fig. 1: problem definition and potential solution, tool development and design, demonstration, and evaluation. We further describe these in detail below in the context of this study. The study was completed in various phases, the first involving a series of exploratory interviews and a short survey conducted between January to June 2021. This was followed by the phases of tool development and evaluation through a series of demonstration workshops from February 2022 to August 2023.

### 3.1 Problem definition and potential solution - Exploratory interviews and short survey

First, the exploratory phase consisted of a set of 24 semi-structured interviews and a short qualitative survey with 16 business professionals from a range of industries. The participants were asked about the characteristics that would be most important to them in an environmental impact measurement tool during their business experimentation phase. The participants were identified through purposive sampling of business model innovators in the authors’ professional networks. The initial participants were chosen because of their involvement in circular business model experimentation as well as environmental impact assessment within sectors that have a high impact reduction potential; furniture, clothing, mobility, energy-using appliances, food and consumer goods. Further participants were recruited using a snowball method, asking each initial participant to identify potential interviewees. Table 1 shows the list of participants and their organisational attributes. A list of the questions asked in the semi-structured interviews and short survey can be found in Appendix A. All responses collected were anonymised and analysed through high-level qualitative coding. This information informed the tool development process and the creation of the first version of the tool. These findings are further detailed in Section 4.3.1.

**Table 1:** List of interview participants and their organisational attributes.

Participant no.	Industry sector	Role of participant	Organisation size	Data type
1	Furniture	Service circular business designer	Large	Interview
2	Furniture	Circular service business designer	Large	Interview
3	Furniture	Project leader of circular supply chain team	Large	Interview
4	Furniture	Sustainable business developer	Large	Interview
5	Energy-using appliances	Co-founder	Small	Interview
6	Energy-using appliances	Co-founder	Small	Interview
7	Energy-using appliances	Director, circular solutions & sustainability affairs	Large	Interview
8	Energy-using appliances	LCA Expert	Large	Interview
9	Food/Energy-using appliances	CEO	Small	Interview
10	Food	Global content creator	Large	Interview
11	Food	Global sustainability manager	Large	Interview
12	Food	Sustainability manager	Medium	Interview
13	Food	Consultant	Small	Survey
14	Food	Technical lead	Small	Survey
15	Mobility	Head of sustainability	Large	Interview
16	Mobility	Co-founder	Large	Interview
17	Mobility	Environmental impact assessor	Large	Interview
18	Mobility	Industrial PhD fellow	Large	Survey
19	Clothing	Founder	Small	Interview
20	Clothing	Marketing & social media manager	Small	Interview
21	Clothing	Director resale ventures & corporate development	Large	Interview
22	Clothing	Head of circular business model incubation	Large	Interview
23	Clothing	Sustainability manager	Large	Survey
24	Consumer goods	CEO & co-founder	Small	Interview
25	Consumer goods	CEO, founder	Small	Survey
26	Construction	Community affairs officer	Medium	Interview
27	Construction	Sustainability manager	Small	Interview
28	Consultancy	Impact entrepreneur & designer	Small	Interview
29	Consultancy	Lead service designer	Small	Interview
30	Consultancy	Climate impact consultant	Small	Interview
31	Consultancy	Senior consultant	Large	Interview
32	Consultancy	CEO & founder	Small	Survey
33	Consultancy	Founder/Director	Small	Survey
34	Consultancy	Founder	Small	Survey
35	Consultancy	Owner	Small	Survey
36	Consultancy	Analyst	Large	Survey
37	Consultancy	Engagement manager, circular design programme	Large	Survey
38	Consultancy	Senior consultant	Large	Survey
39	Consulting	Project manager	Small	Survey
40	Manufacturing	Manager, product stewardship	Small	Survey

### 3.2 Tool development

**Fig. D.1:**
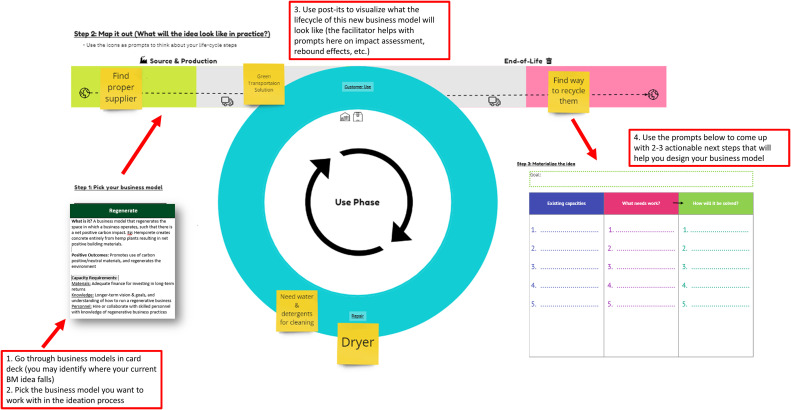
First version of the Circular Rebound Tool.

**Fig. D.2:**
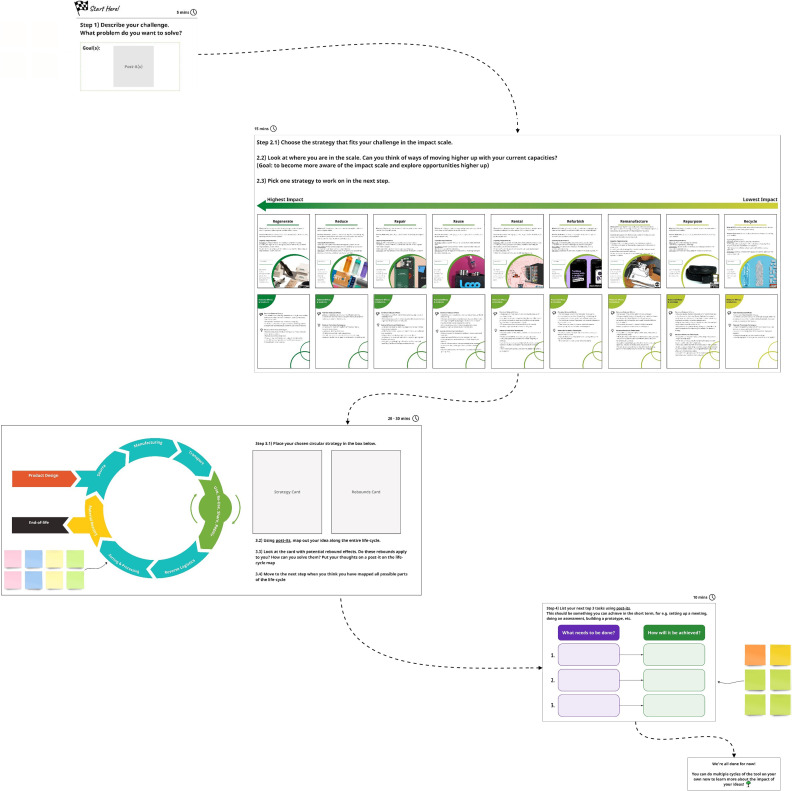
The various steps of Circular Rebound Tool.

The identified research gap was addressed by developing a first draft of a design-ideation tool with the goal of guiding practitioners in making better environmental impact-based decisions in the experimentation phase of their new circular business ideas. This was developed based on triangulation of findings from research gaps identified in literature and from insights discerned from the exploratory interviews and a short survey. The three guiding concepts of the tool development were 9R framework of waste hierarchy, reduction of rebound effects and the life cycle perspective (Kirchherr et al., 2017; Ness et al., 2007; Zink and Geyer, 2017). This first version (Figure D.1 in Appendix D) then underwent iterative testing and evaluation, and was improved through four iterations. These are described in Section 4.3, and the final tool is presented in Figures D.2 and D.3 in Appendix D.

### 3.3 Demonstration workshops

The utility of the tool was tested and improved through a series of 15 iterative workshops. These workshops were conducted in three phases: first, 2 workshops with academics; second 6 workshops with sustainable business design consultants; and third, 7 workshops with employees from businesses that were ideating towards circular business models. In the first two phases, the tool was tested using hypothetical business cases that the participants prepared. In the third phase of testing with companies, the tool was tested on real-world ideas that the companies were experimenting with. After the first phase of testing with academics, the authors conducted a brainstorming session on how to better improve the different elements of the tool. The second and third phases of workshops were conducted parallelly, to better accommodate the schedules of practitioners. In total, the tool was tested with 26 participants. The list of workshops and participants can be seen in Table 2.

**Table 2:** List of workshop participants.

Sr. no.	No. of participants	Role, organisation	Sector	Organisation size	Workshop type	Time taken to use tool (hh:mm)
1	1	Master's student, Lund University	Academia	–	In-person	01:00
2	2	PhD Researchers, Maastricht University	Academia	–	In-person	01:15
3	1	Co-founder, HOMIE	Business – Energy-using appliances	Small	In-person	01:00
4	1	Impact Assessment Business Area Manager, Cleantech Scandinavia	Consultancy	Small	Online	00:23
5	1	Founder & Creative Director, Punctuate Design Studio	Consultancy	Small	Online	00:20
6	2	Director Strategic Innovation + Sustainability Expert, INDEED Innovation	Consultancy	Medium	Online	00:45
7	1	Founder, Candour Digital	Consultancy	Small	Online	00:32
8	1	Project Manager Circular Economy Umwelttechnik BW GmbH	Consultancy	Medium	Online	00:25
9	2	Sustainability experts, reynaers aluminium	Business – Construction	Large	Online	00:23
10	2	Co-founders, ComfyHand	Business – Medical Equipment	Small	Online	01:00
11	1	Partner & Designer, Reform GmbH	Consultancy	Small	Online	00:32
12	1	Head of resell platform of footwear company	Business - Apparel	Large	Online	00:20
13	5	Sustainable business model innovators, Global beauty, haircare & consumer goods company	Business – Consumer Goods	Large	Online	01:05
14	3	CE Specialists, Head of R&D, BayWa r.e.	Business – Solar Power	Medium	Online	00:46
15	2	Circular Business Development Manager & Intern, VAUDE	Business - Outdoor Apparel	Large	Online	00:38

### 3.4 Evaluation of usefulness

In order to improve and develop a useful artefact, continuous evaluation is conducted during the demonstration phase of DSR (Peffers et al., 2007). The goal is to continue with demonstration until no new significant feedback for improvement was forthcoming – the point of saturation. To this end a subjective evaluation was conducted at the end of each workshop. Participants were asked to score the workshop on its ease of use and perceived usefulness (Davis, 1989), as a guide to the development of circular business models with better environmental impact potential and its ease of use, on a five-point Likert's scale. The method of evaluation was based on the Technology Acceptance Model developed by Davis (1989). This model was used as it helps improve the user experience, predict user acceptance of the tool, and identify important factors for acceptance of the tool. The questionnaire for the evaluation can be found in Appendix B. The tool evaluation and improvement process based on feedback is further documented in Table 4. Based on the feedback received, the tool underwent 4 distinct iterations of changes, until no more feedback for major changes was received. This final version of the tool was tested in seven workshops.

## 4 Results: Tool development, description & evaluation

The results of this study are presented based on the recommendations of Gregor & Hevner (2013) on structuring a DSR study that contributes a design artefact. This includes a description of the designed artefact – in this case the tool, the artefact development process, and an evaluation of the usefulness of the artefact among its intended users. In accordance, we first present the final tool, describe its key concepts, and provide guidance on how to use it. We then detail how the tool was developed throughout this study and describe changes made based on the feedback from the workshop participants (Gregor & Hevner, 2013).

### 4.1. The Circular Rebound Tool

**Fig. 2:**
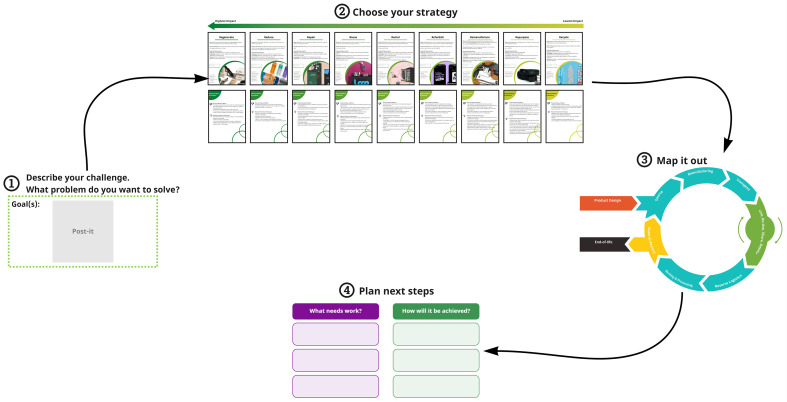
A snapshot of The Circular Rebound Tool.

The steps of the Circular Rebound Tool are displayed in Fig. 2. Based on the exploratory interviews and a qualitative survey, we designed the tool so that it will be easy to use and enable the users to make judgements with limited data, while nudging them to make quick and iterative changes to their ideas by incorporating rules of thumb (Das et al., 2022). The tool has four steps: 1) a description of the challenge, 2) choosing a circular business strategy from a set of cards that each describe a circular business strategy, and the potential rebound effects of the strategy and ways to potentially avoid them, 3) mapping of the idea along the complete life cycle of the business model and 4) planning of next steps. The tool is based on three important concepts: the zero-waste hierarchy (Kirchherr et al., 2017), the avoiding of rebound effects (Metic and Pigosso, 2022; Zink and Geyer, 2017), and a life-cycle perspective (Ness et al., 2007). We describe each of these key concepts and how they have been integrated into the tool below, before detailing the steps to guide users through the tool.

The first concept the tool is based on is the zero-waste hierarchy. It allows users to rank the environmental impact reduction potential of the circular business strategies that they want to employ. We used Kirchherr et al. (2017)’s “9R Framework” to conceptualise this. The framework ranks different circular business strategies based on their environmental improvement potential. These strategies — refuse, rethink, reduce, reuse, repair, refurbish, remanufacture, repurpose, recycle, and recover — are ranked from highest to lowest impact reduction potential. For example, the circular business strategy of ‘Recycle’ ranks lower than ‘Reduce’. This is because most ‘Recycle’ approaches involve downcycling of materials over time, as the quality of materials reduces with each recycling cycle. Whereas ‘Reduce’ calls for a more absolute reduction in use of resources and materials in manufacturing. A more simplified version of this hierarchy — reuse, reduce, recycle — is commonly represented as an inverted pyramid of the zero-waste hierarchy geared towards consumers (Zhang et al., 2022). We used the 9R framework as a starting point, but then later settled for a new version based on feedback and brainstorming from the evaluation phase, with 9 cards that each represent one of the following circular business strategies: Regenerate, Reduce, Repair, Reuse, Rental, Refurbish, Remanufacture, Repurpose, and Recycle. These changes were made after discussion amongst the authors. A new strategy, ‘Regenerate’ was added to broaden the range of strategies beyond just focusing on harm reduction, and moving towards strategies that are ‘net positive’ in their environmental impact. Meanwhile, refuse and rethink were merged into 'Reduce’, as that captures the core concept of all three strategies. The strategy of ‘Rental’ was included as it differs from both reuse and refurbish and is a very common business model, for example in product-service systems.

**Fig. 3:**
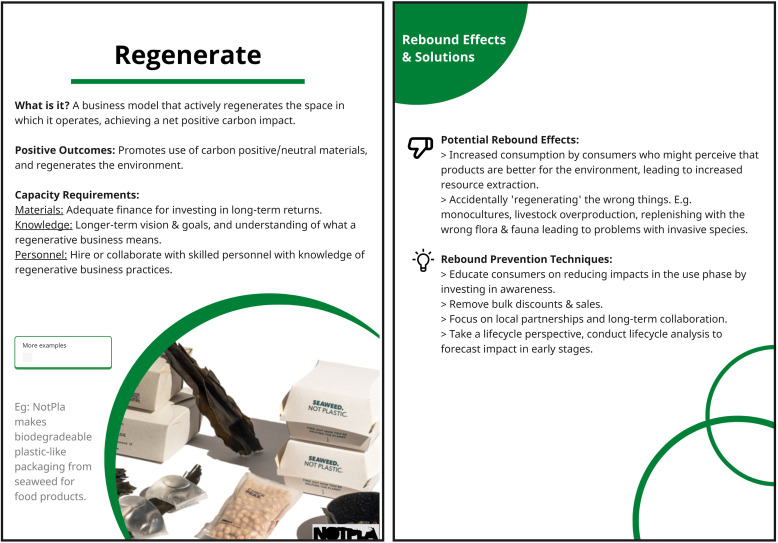
An example card with information on the R strategy of ‘Regenerate’, and its corresponding rebound effects and potential prevention techniques.

**Fig. D.3:**
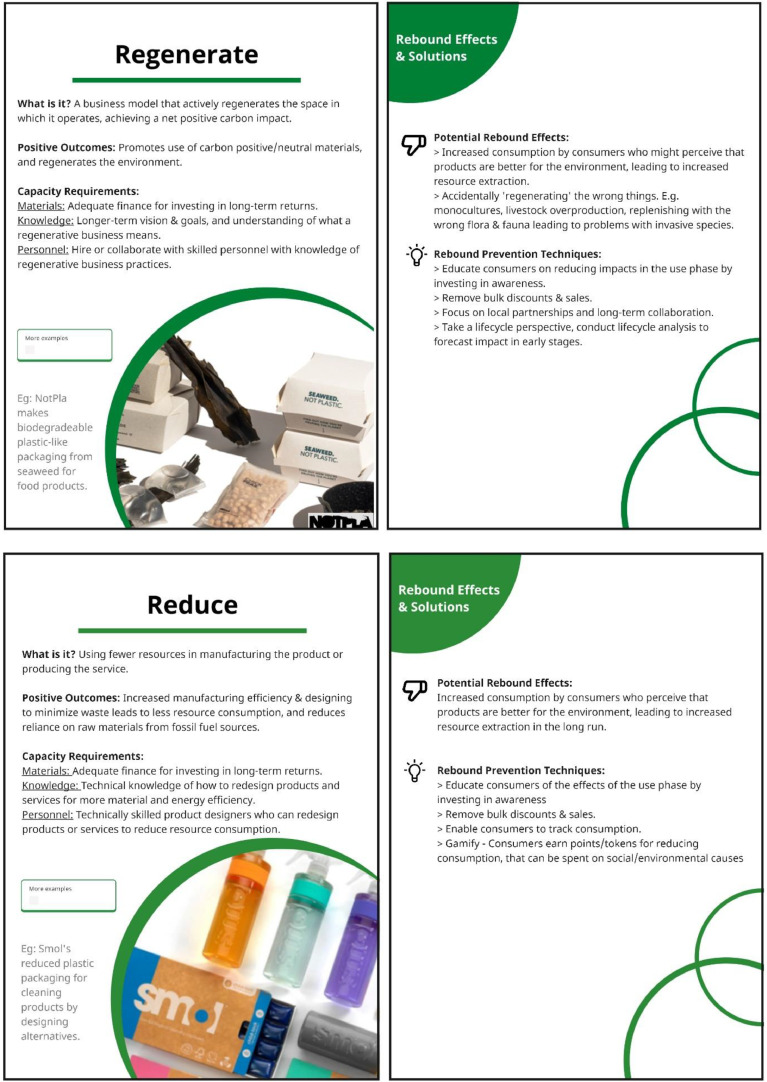

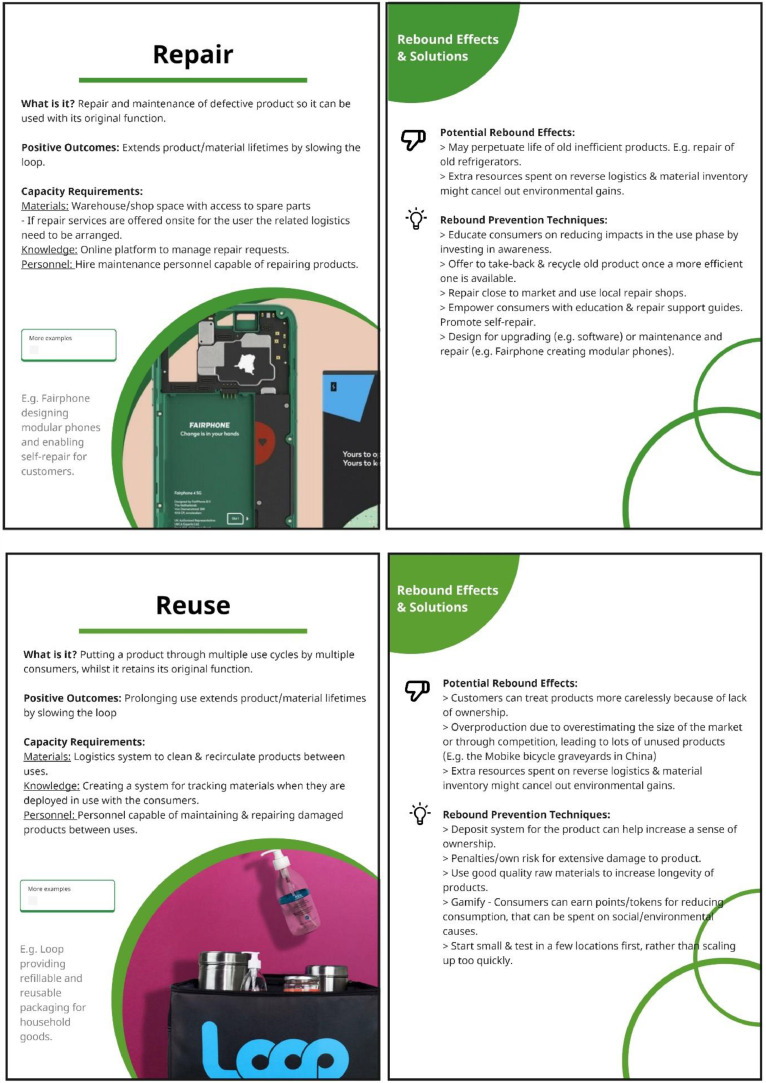

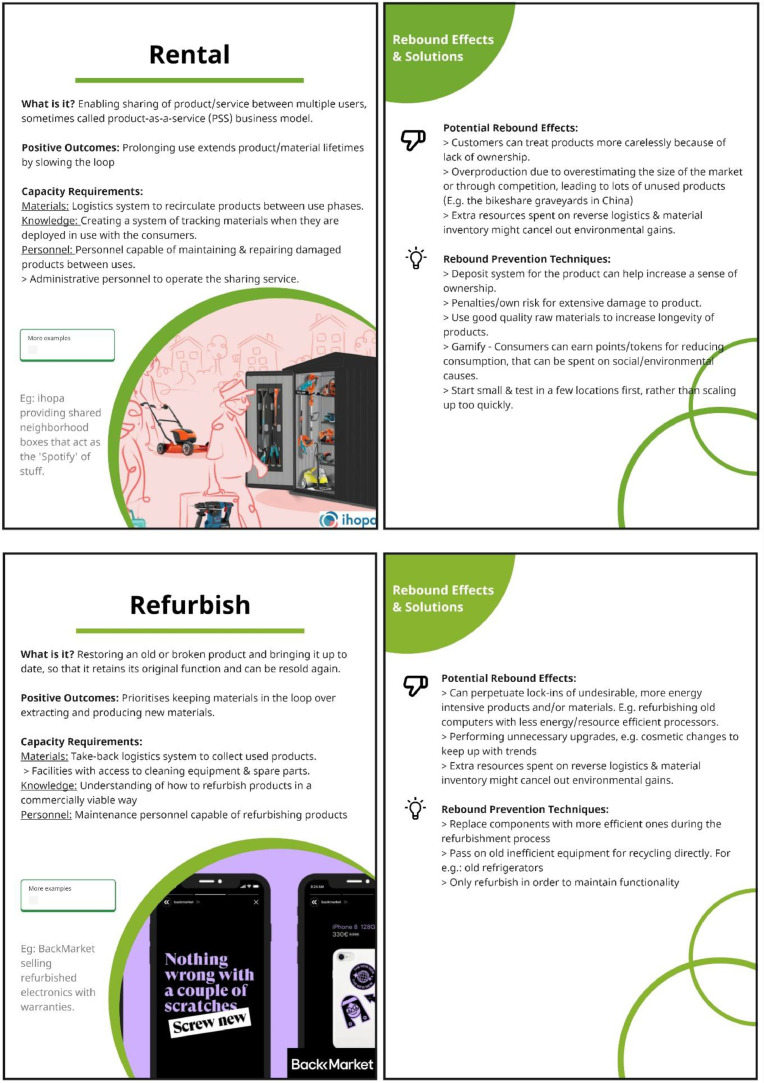

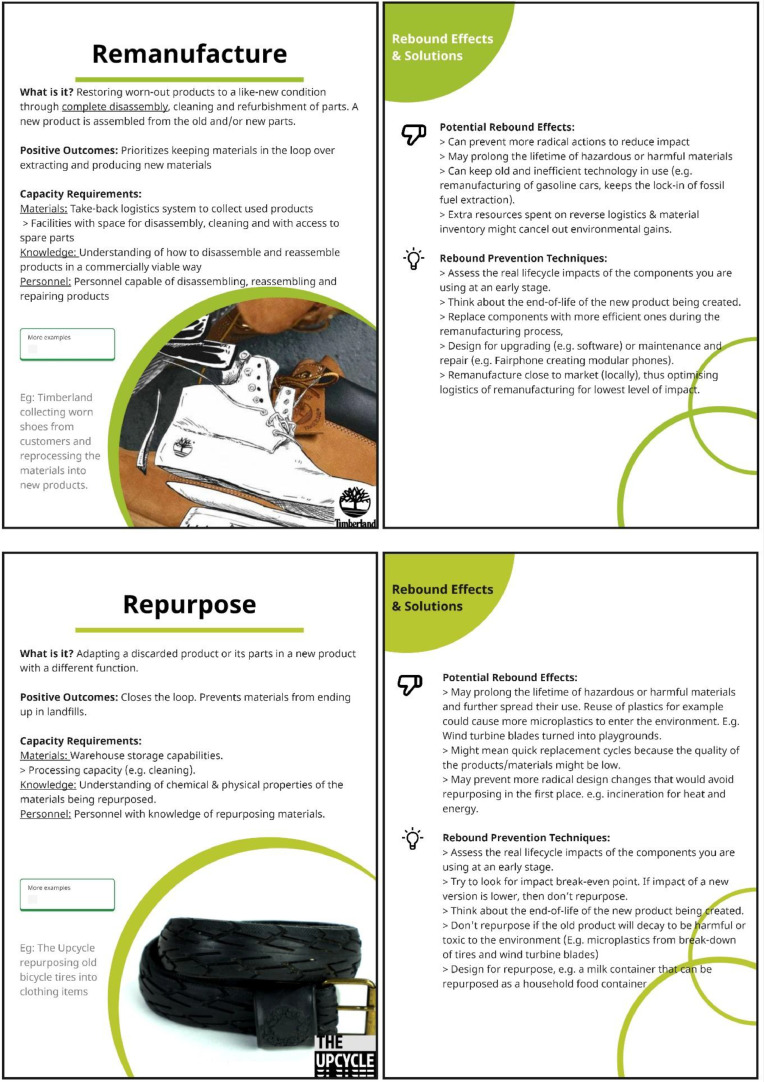

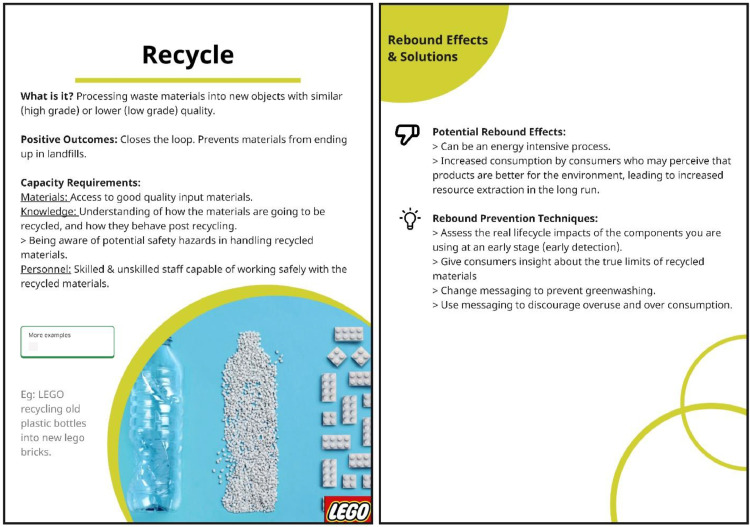
The circular business strategy cards arranged according to environmental impact reduction potential as used in Step 2 the tool.

Each card in Step 2 contains one R strategy, a description of what the strategy is, it's expected positive outcomes (e.g.,reduced emissions or raw material utilisation, or regenerative effects) the basic capacity requirements needed to realize it, and examples of companies that have been implementing the strategy in practice. An example is shown in Fig. 3. The full set of cards are shown in Fig. D.3, Appendix D.

**Fig. 4:**
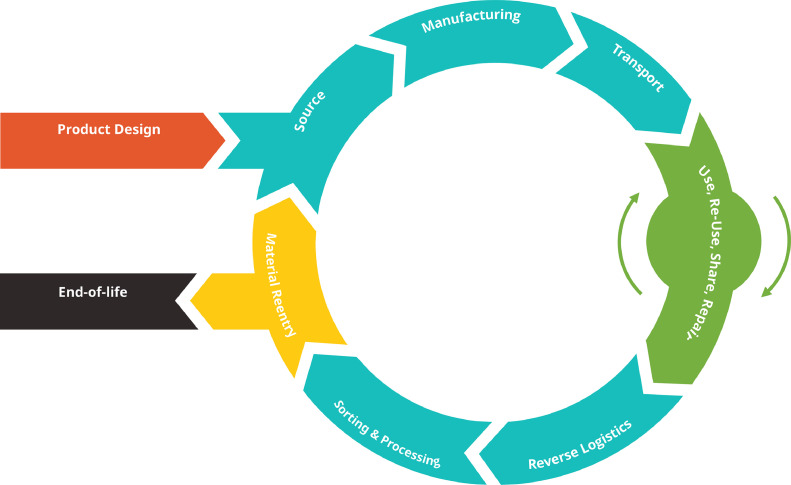
The life-cycle map diagram used in Step 3.

The second concept included in the tool was rebound effects of the different circular business strategies, and potential ways to avoid them. This is because, as discussed in Section 2, they can extensively influence the net environmental impact of circular business models. Each card from Step 2 contains information on potential rebounds that can result from choosing a particular R strategy, as well as potential ways to avoid the rebounds. An example is shown in Fig. 4. The full set of cards can be found in Fig. D.3 in Appendix D. The focus here is on the micro-level direct rebound effects, as these are what are within the zone of control of the companies in the experimentation phase. These need to be considered first before moving on to more abstract indirect rebound effects. We used inspiration from these two theories - Kirchherr et al.(2017)’s 9R framework and Zink & Geyer (2017)’s concept of circular economy rebound - as the starting point of conceptualising Step 2 of the tool. While these theories have slightly differing conceptualisations of circular strategies, for the purposes of the tool's design they were considered to be complimentary. To the best of our knowledge, this was a first attempt at creating a framework for classifying rebound effects of different circularity strategies. So, we base these ‘rebound cards’ on a brief review of rebound effects studies (e.g., Catlin and Wang, 2013; Johnson and Plepys, 2021; Niero et al., 2021; Siderius and Poldner, 2021; van Loon et al., 2021; Warmington-Lundström and Laurenti, 2020; Zink and Geyer, 2017). The framework for the classification of rebounds and corresponding mitigation strategies was then arrived at through an iterative brain- storming session between the authors. A systematic literature review was not done, as this study followed the DSR method, and such a review was out of scope of the study.

The third component of the tool is a life cycle perspective on the product or business model idea, while designing it in the early stages. This can give crucial foresight about the environmental outcome of the business model idea in all stages from cradle-to-grave/cradle. This is represented in Step 3, where a figure lets the user map ideas along the complete life cycle of the product using post-its (Figure 5).

### 4.2 How to use the circular rebound tool

The final Circular Rebound Tool involves a **4-step process** that aims to guide experimentation towards business models with lower environmental impact potential. A more detailed contextual snapshot of the different steps of the tool, and their corresponding instructions in the brainstorming canvas, can be found in Appendix D (Figures D.2 and D.3). A brief overview of the process can be seen below:

*Pre-work:* In Miro, the participants are first given a quick tutorial on how to use the software, and an example case of using the tool. This serves the purpose of allowing participants to have a more productive user experience.

*Step 1:* In the first step, participants define their circular challenge, or the problem they are trying to solve.
*Step 2:* Participants then go through the hierarchy of circular strategies (Figure D.2 & D.3, Appendix D), and identify the ones that would be the best solution for their challenge or existing business idea. Then they are prompted to explore where their business idea lies in the hierarchy of environmental impact reduction potential, and whether they can go higher in their experimentation based on the capacities they have. After this, participants are prompted to pick one or more strategies to work on in the next step. The workshop gets more focus from picking one strategy, but more than one strategy can be used, in later stages, as there can at times be overlaps of different strategies in one business model idea.
*Step 3:* The chosen strategy, and its corresponding rebound effects cards are then placed in the designated box. Participants are then asked to map out what their business idea can look like in its entire life cycle using post-its. The instructions then prompt the participants to observe what the potential rebound effects of their idea can be, and think about how they can avoid them. They are then asked to put these thoughts down on the life-cycle map.
*Step 4:* Finally, participants are asked to define their next steps concretely. They are asked to list out their top three short-term priority tasks after using the tool, and how they will achieve them. This aims to make the results obtained from the brainstorming process more actionable.

### 4.3 Iterations of the tool and changes made throughout the workshops

The tool development happened first through exploratory interviews and a short survey and second through, consecutive tests in three phases with three participant groups: academics, designers & consultants, and businesses. The three sections below describe this in more detail. Table 3 documents the different changes the tool went through during its four iterations along with the corresponding rationale for the changes. The complete list of feedback received during the workshops can be found in Table C.1, Appendix C.

**Table 3:** List of improvement points based on feedback received during the testing and evaluation of the tool through the workshops.

#### 4.3.1 Exploratory interviews & short survey

The first step involved developing a prototype version of the tool based on insights from the exploratory interviews and short survey to better meet the needs of practitioners. The insights from the exploratory phase revealed that practitioners were seeking an easy-to-use tool that would allow them to make judgements about the environmental impact with limited data, and that could be applied rapidly and iteratively in the experimentation phase. They were also looking for tools that could guide them towards better business model decisions, and where a change in their business model would create the highest impact, instead of just providing data on CO_2_ emissions. This quantitative measurement was deemed less useful by the practitioners while in the experimentation phase. We triangulated these insights from the exploratory interviews and survey with insights from literature to determine that a qualitative tool that incorporates rules of thumb with a full life cycle perspective would be the most useful starting point. Thus, the first version of the tool incorporated the concepts of life-cycle assessment, the zero-waste hierarchy, and avoiding rebound effects.

#### 4.3.2 First testing phase with academics

The academics trialled the first iteration of the tool with hypothetical business cases, in in-person workshops. It was observed that high levels of intervention and guidance were required from the facilitator. Thus, in the following iteration, the instructions corresponding to the different steps were improved. The tool was also moved online into the Miro whiteboard software to allow for better functionality, facilitation, and use with workshop participants in multiple countries.

#### 4.3.3 Second testing phase with designers & consultants

The designers and consultants also used hypothetical business cases to test the tool. Their inputs were considered important as the chosen practitioners worked in design firms that typically assisted companies in redesigning their business models or innovating new ideas. It was observed during the workshops that more clarification was still required about the different elements and steps of the tool. This was improved upon in the third iteration. Further, it was observed that there was a need for more space for the participants to interact with the tool, so a new separate brainstorming space was added.

#### 4.3.4 Third testing phase with businesses

The practitioners from businesses used their real-life cases while testing the tool. It was observed that more examples of businesses that were already trialling the circular business strategies in different sectors needed to be added, to make the tool more relatable to a wider range of users.

#### 4.3.5 Evaluations and tool iterations

In their evaluations of the tool participants gave the tool's usefulness an average rating of 4.3/5, and a rating of 4.2/5 for ease of use. Participants noted for example that the Circular Rebound Tool helped them consider rebound effects like they never had before: “*Rebound effects framework! It was brilliant in having a holistic view on ideas.*”, and that it helped provide structure to their brainstorming process and an “*Opportunity for constructive conversations around circularity with [their] partners*”. Participants also noted that the tool was *“really easy to use even though it took into account some of the more complex concepts such as LCA/Waste Hierarchy etc.”* and *“That it can be relatively easy to draft out a circular business model concept, as long as it is guided by the right tooling & models provided in the workshop”*.

Further, it was observed most participants (11 out of 15 workshops, i.e., 73 %) tried to engage with business models on the higher environmental impact improvement end of the scale. This could be considered to be an indicator that users were successfully being nudged towards circular business ideas with higher environmental impact reduction potential.

## 5 Discussion

This study makes two main contributions. First, it provides a new tool that can support circular business model innovators towards strategies that have higher environmental impact reduction potential. Past research found that business innovators do not use detailed environmental impact measurement methods in the early experimentation phase. Instead, they resort to rules of thumb as they are easier to use (Das et al., 2022). Further, most business model ideation tools do not factor in rebound effects and thus, do not take the final environmental impact into account (Castro et al., 2022; Pieroni et al., 2019). Our tool bridges this gap in business model experimentation research (Blomsma and Tennant, 2020; Bocken et al., 2016a; Walzberg et al., 2021), and the findings can be useful for innovators, consultants, academics and businesses that are looking into the transition to circular business models. While our tool was specifically designed for the experimentation phase, we believe its use could be extended to the entire business model ideation phase. Past research has also shown that many good tools that are developed in academia are not used in practice (Baumann et al., 2002; Tyl, 2015). To counter this the tool was developed with extensive insights from practice, based on past research (Das et al., 2022), exploratory interviews, a short survey, and workshops, to ensure a higher rate of uptake in practice. The tool will also be made freely available as a template in the Miroverse software, to allow for it to be used by business innovators.

Second, in a theoretical context, previous studies have investigated the topic of rebound effects within the circular economy (Castro et al., 2022; Figge and Thorpe, 2019; Metic and Pigosso, 2022; Zink and Geyer, 2017). Zink & Geyer (2017) were the first to classify these effects, while Castro et al. (2022) and Metic & Pigosso (2022) have extended this work by identifying gaps in existing research and establishing a research agenda for the field. Most recently, Zerbino (2022) has proposed a conceptual methodology for managing rebound effects in circular systems that includes anticipating these effects, monitoring impact, designing policies, and engaging stakeholders. This study goes a step further in providing a first attempt at developing a framework to map rebound effects for different circular business strategies, and potential pathways to prevent them. This was a significant research gap that was identified by previous studies (Castro et al., 2022; Metic and Pigosso, 2022). This can provide a foundation for further research and practical guidance in developing more sustainable circular business models, whose benefits are encumbered by rebound effects. Most businesses tend to be unaware of the potential of being faced with rebound effects that offset their planned environmental gains from their new circular ideas. Thus, we see this tool as having an educational purpose for practitioners in this regard.

### 5.1 Limitations & future research

This study has a few limitations. First, it lacks quantitative measurement of the environmental impact of the business ideas and the tool does not specify the types of environmental impacts (e.g., waste, emissions) but rather discusses them at an aggregate level. This limitation was recognised early on, however, the main aim was to provide evidence-based qualitative guidance on environmental impact during the ideation phase, before data can be gathered to conduct more detailed assessments. This is based on findings from past research that showed that practitioners tend to avoid using complicated, time-consuming environmental impact assessment methods in the experimentation phase, and instead rely on rules of thumb to make decisions (Das et al., 2022). Future research can explore the integration of simpler quantitative measurements with qualitative ideation tools.

Second, the tool also lacks a section to determine the practical feasibility of the business model ideas that are tested in the workshops. This was excluded to avoid further complexity in the tool, and also because many such tools exist already to help make feasibility-based decisions of business ideas (e.g., The Upstream Innovation Tool by the Ellen MacArthur Foundation, Life-Cycle Analysis by The Green Sprint). Further, the framework for the classification of rebounds was not a result of a systematic literature review, because – as mentioned in Section 4.1 – this was beyond the scope of the chosen method. We also acknowledge that not all the rebound prevention strategies listed in the cards might be within the scope of control of any single company. Future research can address the classification of rebounds by conducting a systematic literature review.  This could also address what mitigation strategies are actionable by companies, or perhaps by mutual interest groups who have the resources and opportunity to affect change in ways that individual organizations do not.

Third, the evaluation of the tool only focused on the ease of use and perceived usefulness of the tool for users, rather than the effect on reduced environmental impact in real world applications. As the purpose of the study was to evaluate an ideation tool, the long-term impacts of the application of the tool were beyond the scope of this study. Once disseminated and used, future research should seek to address the impact of the tool.

Lastly, the tool was mostly tested with small to medium sized organizations - only 4 out of the 13 company workshops took place with larger companies. This might make the tool less well adapted and relevant to application within large organizations. However, the workshops with larger organizations did not indicate issues of scalability, suggesting that the tool could very well scale up to application within larger organizations.

## 6 Conclusion

In conclusion, this research aimed to explore if a tool can be created to support practitioners in making business decisions that lower environmental impact through promoting conscious thinking about preventing rebound effects, while they are experimenting with new circular business ideas. In investigating this aim, we used the design-science research methodology to empirically test and improve The Circular Rebound Tool through 15 iterative workshops. The first step in this was to conduct a series of exploratory interviews (*n* = 24) and a qualitative survey (*n* = 16), to explore what practitioners that are ideating new circular business models need from an environmental impact assessment tool in the experimentation phase. This was followed by a series of 15 workshops where the tool was demonstrated, evaluated and improved to give the final Circular Rebound Tool.

As companies navigate the increasingly uncertain and risk-ridden business landscape, they must keep multiple factors in mind while designing new business models that have a net positive environmental impact. As a result, tools are needed that can support them in making environmental impact-based decisions while they are still in the experimentation phase where a lot of future impacts can get locked-in. To fill this gap, The Circular Rebound Tool incorporates the principles of the waste-hierarchy, the life-cycle assessment and stimulates conscious thinking about reducing rebound effects, to give guidance on the environmental impact of circular business models. This can allow practitioners to use a more evidence-based and structured method to be better informed about the environmental impact of their ideas, instead of resorting to ‘rules of thumb’ (Das et al., 2022), which can oftentimes be inaccurate.

Future research could include longitudinal studies that follow business ideas resulting from the tool, to see whether the planned environmental impact reduction potential actually translates into practice. Another avenue of research could be to investigate differences in how the tool scales and performs between small, medium and large organisations. Future research could also investigate the effect of any positive rebound effects on environmental impact of circular business models, and how these might be incorporated at the business model ideation stage.

## Data Availability

Further data on the specific workshop participants and outcomes is not available. However, the primary outcome of the study, and the workshop process, the tool, is made available. The Circular Rebound Tool template can be accessed freely in the Miroverse under the template name “The Circular Rebound Tool”. An offline version of the tool can be viewed in Appendix D of this paper.

## CRediT authorship contribution statement

**Ankita Das:** Conceptualization, Visualization, Methodology, Investigation, Data curation, Writing – original draft, Writing – review & editing. **Jan Konietzko:** Conceptualization, Visualization, Writing – review & editing, Supervision. **Nancy Bocken:** Conceptualization, Visualization, Writing – review & editing, Supervision, Funding acquisition. **Marc Dijk:** Conceptualization, Supervision.

## Declaration of Competing Interest

The authors declare that they have no known competing financial interests or personal relationships that could have appeared to influence the work reported in this paper.

## Figures and Tables

**Table C.1:** Full list of feedback received from the demonstration and evaluation workshops.

Workshop No.	What did you like most about the tool/workshop? What was most useful?	What was difficult? What did you feel needs improvement?	What did you learn?
Phase - 1			
1	*“Picking business model was easy, the tool helped to narrow down the possible options well”* *“From design perspective - Helped think about what specifically needs to be changed to make product more sustainable”* - Liked overall process	*“More help needed with some of the prompts related to life-cycle perspective. So add questions like: how do you imagine your product will move through its lifecycle?* *What will be needed at every step?* *How would model the average lifecycle use?”* - Highlight the impact points better - Did not understand the current illustration of the tool very well right now	<Question not asked in this phase>
2	*“I was able to think about different options that are even more environmentally friendly”* *“It's very good to reflect on the rebound effects.”*	*“Lay people might not understand all the terms”* *“Terminology”* *“Some examples might be hard to understand (very context specific)”* *“The icons might be hard to understand”* *“Better name for circle in Step 2″* - Have a clearer scale (highest to lowest) - Clarify descriptions a bit more - Make it less text heavy - Repair should come before reuse - Make examples easier to understand - Have a section for sourcing & production - Workshop would be better if done in one group/case study, instead of two separate ones - Use of post-its always, instead of writing on actual template - Create bullet points	<Question not asked in this phase>
Phase – 2 & 3			
3	*“The visualisation - Very nice!”* *“Useful: The ways to avoid rebounds”*	*“Mapping my value chain & business model”* *“More tips on how to avoid rebounds”* - Add one example per sector - ‘Sharing’ strategy needs to better represent PSS types of models	<Question not asked>
4	*“The explanation of the life cycle approach and the design for circularity process. It was very clear and it is a great setup for the work ahead.”* - Life-cycle approach explanation was good.	*“It was fairly simple. However, I miss a section that considers the feasibility of the actions identified for the survival of the business. Should they establish a hierarchy of actions, what should be done first, what has more impact considering the effort that it requires?”* - Problems with locking & grouping of the different Miro board elements - Change spacing of life cycle map - Conduct workshop in Meeting mode - Make step relations clearer - Use of post-its for Step 3 - Establish hierarchy/urgency in Step 3 - Have room for reflection on the feasibility of actions	*“I learn how to structure the logics of circular economy. Really interesting!”*
5	*“Easy and quick!”* - 'What to expect?' Intro to workshop was great. A lot of tools don't have easy to understand instructions at the start - Next steps - Step 4 was good to make things more concrete - Helped to understand what priorities should be	*“There is a lot of information on each page, it will be easier to have 1 instruction per page in small and the action part bigger on the page. And user needs to know a little already about LCA and the circular strategy.. if not, it will take a lot of time to read and understand each strategy”* - One sentence description of strategies would be good - Life-cycle map could be circular - Larger brainstorming space needed	*“Thanks a lot [researcher's name] for sharing this workshop!”*
6	*“Rebound effects framework derived from CE strategies, it triggered more ideas.”* *“Taking a look to REBOUND effects as this is often ignored with only looking at the benefits”* - Really loved the rebound effects aspect. Never seen it before - Got them thinking about recycled materials - Flow was easy (low intervention by researcher) - Would like to have access to the board for future use/brainstorming	*“More guidance in mapping out the Life Cycle to what to do next. Maybe there is time boxing if used in workshop setting.”* *“To me especially the business impact side was missing -this is a crucial factor imagine circular transport or additional energy necessary for recycling”* - Keep the cards open, so multiple people can view them at the same time - Better life-cycle map. Clearly mention the different stages more - Improve instruction prompts to make greener like "What could you do to…?" - Make Step 4 ranking more clear - Fix some spelling mistakes in the cards - Add more case study examples & a bit more clarity	*“Rebound effects framework! It was brilliant in having a holistic view on ideas. With ideas we tend to see it as a whole simply because it is new, rebound effects served as a good reflection tool to make them complete.”* *“It made obvious what data sources are necessary and/or that a principle needs iteration to come to a solution (if recycling seems to be the right way you may recognize it is not working and you need to change)”*
7	*“The fact that it was really easy to use even though it took into account some of the more complex concepts such as LCA/Waste Hierarchy etc.”* - Liked the simplicity - Was holistic & easy to use - High-level & usable	*“I feel like product design & its potential impact could be included more”* - Include a ‘Design phase’ in the lifecycle map - Also look at supply chain + collaboration - Convert ‘Production’ to ‘Manufacturing’ in Step 2 for more clarity - Mention that the final step is like a to-do list.	*“That it can be relatively easy to draft out a circular business model concept, as long as it is guided by the right tooling & models provided in the workshop”*
8	- Found it easy to use. No big questions during use	- Make the ‘Transport’ step in the lifecycle map clearer - Reword Step 4 for better clarity: What do you need to do to make your idea a reality? - Add numbers to Step 4 - Make prompt instructions more brief - Add a concluding message at the end of the tool	<No response>
9	*“Attention to the rebound effects”* *“Attention on the rebound”*	*“Choosing the R strategy was difficult”* *“Define tasks on short term”* - Link Goal Step to Step 2 with a sentence - Make clearer question prompts in Step 3 - Turn rebound effects pop-up box into card, since 2 people cannot see it at the same time - Steps 3 & 4 need to have end instructions to prompt for the next step	<No response>
10	*“Opportunity for constructive conversations around circularity with both partners”* *“Information about the different stages of circularity.”* - Easy to use, really liked it - Would like to have access to the board for future use/brainstorming	*“Don't know”* - Make questions more specific - Step 4 change size of boxes to be more intuitive - Add a time indicator for how much time should be spent on each step	*“To consider rebound effect”* *“A structure to structure my thoughts about a circular business model”*
11	*“Mapping my ideas and decisions on the life cycle.”* - Lifecycle mapping was great - Very easy & simple to use, no such big barriers to use - Informs the user very well	*“This tool is probably most helpful for fast moving consumer goods or B2C. Considering other industries and businesses would be helpful.”* - Didn't really fit the specific business case of creating a sustainable value chain for leftover scenery backdrops of a dance house - Was not able to dig deeper into the strategy/rebound effects in the short time span of the workshop - Rewrite Step 4 instruction for clarity: "What needs to be done?" - For Step 1 - Make clearer what challenge can look like	*“That there is a simple tool to evaluate ideas regarding their sustainable impact and possible rebound effects within the ideation phase.”*
12	- Much-needed idea - A lot of lack of knowledge on implementation - A good start	- The Step 2 impact scale can be made more obvious/clear - Add time stamps on the left - Fix grouping issue with cards - Fix some typos & grammar errors - simplify language in the text - Add more specific examples for different industries	<No response>
13	*“Gave some ideas on how to solve a problem”* *“Shared tool”*	*“The cards are quite generic - so for many niches it might need a little translation to get the right concepts which should be applied”* *“Focus on 1 card only”* - Easier if one person leads a brainstorming session (make this clearer in the instructions) - Move Step 3 time stamp to be more visible	*“I liked the circular economy tool chart, very clear.”* *“Structured tool to think circular economy”*
14	*“To identify the rebound effects and see how we can contribute to a positive process, reducing those effects.”* - Very nice exercise - Good for idea organisation - Liked the emphasis on rebound effects	*“It was not so easy to identify what to put/how to fill the circle picture. Maybe some written examples would have been useful to guide on this part.”* - Add more examples for different industries - Tool not as effective in every sector. Some need facilitator input more than others - Fix some formatting issues - Make clearer that not all rebound effects can apply to every case & make time stamps clearer. - More guidance on financial feasibility would have been nice	*“I could remember the rebound effect concept as well as the importance of identifying and addressing them”*
15	*“Get an overview of rebound affects applicable to the business model.”* *“Cards with possible rebound effect to think of”*	*“Provide more information on what needs to be added in the lifecycle template (per step)”* *“Usability to put all the cards around the life cycle was a bit tricky”*	*“Holistic overview of the business model its rebound effects and option to solve them.”* *“We need more data to continue :)”*

## Data Availability

Data will be made available on request.

## Appendix A

The following is a list of the questions that were asked to the participants of the semi-structured interviews in the exploratory phase of the research. The questions were chosen after discussion and deliberation amongst members of the research team. The questions were chosen based on their apparent relevance to the research question. The questions were sometimes adapted to better suit the context and organisational role of the interviewees.

(1) How do you **currently measure** the environmental impact of your business activities?
(2) When you design a new business strategy or innovation for improved sustainability, **how do you keep track** of environmental impact?
(3) What are the **most important features** of an environmental impact measurement tool for you?

Below is this questionnaire that was shared with the participants of the short survey. The mandatory questions are marked with an asterisk:

### Impact assessment of circular service business models

**Welcome to the Maastricht University study on "Environmental Impact Measurement of Circular Business Models"**

Does 'circular' automatically mean more sustainable? Do companies measure environmental impacts of their circular business models now? If they create new business offerings, then do they assess environmental impacts? More companies are moving towards service-based business models (e.g. subscription, renting, leasing), in order to become more circular and sustainable. We are conducting a research study to understand how they continue to keep track of environmental impacts.

**We would like to invite you to complete the following 5-minute survey, to help us answer these questions.**

**Data Processing**

This survey is part of the European Research Council, funded Circular X project at Maastricht Sustainability Institute, Maastricht University. The data is collected in line with GDPR guidelines. Your company name and role will be anonymized, and the responses will be aggregated and analysed. No personal identifying information will be published. The data is only accessible to the researchers (Ankita Das, Dr. Jan Konietzko, Dr. Nancy Bocken).

Participation is voluntary, and you may refuse to participate at any time. If you have any questions, please feel free to contact: Ankita Das; a.das@maastrichtuniversity.nl

Q1 What ‘industry’ do you work in? ․

Q2 What organisation/company do you work for? ․

Your answer will be anonymized.

Q3 What is your role in the organisation? ․

Your answer will be anonymized.

Q4 Do you measure the environmental impact of your business? ․

Yes (1)

No (2)

Not sure (3)

Q9 What type of tools and methods would be part of an ideal future environmental impact measure for you? ․

Q12 When your company designs new strategies to improve sustainability, do you forecast the environmental impact reductions of these new projects? ․

Yes (7)

No (8)

Other (10)

*Display This Question:*

*If When your company designs new strategies to improve sustainability, do you forecast the environme... = Yes*

Q13 Could you explain how?

*Display This Question:*

*If When your company designs new strategies to improve sustainability, do you forecast the environme... = No*

Q14 Please explain why not.

*Display This Question:*

*If When your company designs new strategies to improve sustainability, do you forecast the environme... = Other*

Q15 Please explain your choice *

Q16 What is needed from your perspective to better measure and forecast the environmental impacts of your business and future projects?

Q17 Would you like to follow-up and stay updated on the research results? Please leave your email address below

## Appendix B – Feedback Form

The purpose of this tool was to guide innovators towards circular business models with higher environmental impact reduction potential, and to inform them about possible rebound effects.

1. The tool was useful to address the above purpose

2. How easy was it to understand and use the tool?

3. What did you like most about the tool/workshop? What was most useful?

4. What was difficult? What did you feel needs improvement?

5. What did you learn from the workshop?

## Appendix C – Workshop qualitative feedback & observations

Below is the full set of feedback received about the tool during the demonstration and evaluation phases of the DSR methodology. The text in quotes is taken directly from the feedback forms the participants filled out. The remaining text is from the researcher's notes on the process and verbal feedback received during the workshops.

## Appendix D – The Circular Rebound Tool

Fig. D.1 showcases the first version of the tool, before it underwent any improvements through iterative testing in the demonstration workshops. The following Figs. D.2 and D.3 show the final versions of the tool.

Figure D.2 below showcases the various steps and instructions of the circular rebound tool as they appear in the original brainstorming canvas. Figure D.3 shows a more detailed view of the content of the cards used in Step 2 to illustrate the scale of environmental impact potential of the various circular strategies. The online template of the tool is available in open access in the Miroverse online environment with the name “The Circular Rebound Tool”.
